# Contextualising the Perceptions of Pharmacists Practicing Clinical Pharmacy in South Africa—Do We Practice what We Preach?

**DOI:** 10.3389/fphar.2021.734654

**Published:** 2021-12-03

**Authors:** Elmien Bronkhorst, Natalie Schellack, Andries G. S. Gous

**Affiliations:** School of Pharmacy, Sefako Makgatho Health Sciences University, Pretoria, South Africa

**Keywords:** pharmacy practice, clinical functions, pharmacy education, pharmaceutical care, clinical pharmacy

## Abstract

The National Department of Health published their Quality Standards for Healthcare Establishments in South Africa and introduced the National Health Insurance, with the pilot phase that commenced in 2012. The system requires an adequate supply of pharmaceutical personnel and the direct involvement of clinical pharmacists throughout the medication-use process to ensure continuity of care, minimised risk with increasing improvement of patient outcomes. The study aimed to provide insight into the pressing issues of clinical pharmacy practice in South Africa, and sets out to contextualise the current profile of the pharmacist performing clinical functions. The study used a quantitative, explorative, cross-sectional design. The population included pharmacists from private and public tertiary hospitals. A questionnaire was administered, using Typeform™. Ethics approval was obtained from Sefako Makgatho Health Sciences University, National Department of Health and Private Healthcare groups. Categorical data were summarised using frequency counts and percentages; continuous data were summarised by mean values and standard deviations. The sample size included 70 pharmacists practicing clinical pharmacy (private sector *n* = 59; public sector *n* = 11). Most participants hold a BPharm degree (busy with MPharm qualification) (64%; *n* = 70). No statistical significance was found between participants in private and public practice. Most pharmacist agreed (32% (private); *n* = 59) and strongly agreed (45% (public); *n* = 11) to have sufficient training to perform pharmaceutical care. The majority respondents felt that interventions made by the pharmacist improved the rational use of medicine (47% (private); 55% (public). Pharmacist interventions influence prescribing patterns (42% (private); 64% (public); and reduce polypharmacy (41% (private); 55% (public). The clinical functions mostly performed were evaluation of prescriptions (private 90%; public 82%), while the top logistical function is daily ordering of medication (40.7%; private), and checking of ward stock (36%; public). Although not all pharmacists appointed in South Africa has completed the MPharm degree in clinical pharmacy, the pharmacists at ward level perform numerous clinical functions, even if only for a small part of their workday. This paper sets the way to standardise practices of clinical pharmacy in South Africa, with a reflection on the differences in practice in different institutions.

## Introduction

In South Africa, there is a marked inequality to medicine access. The WHO states that in South African Health Care, the private sector accounts for 81% of healthcare spending of the gross GPP for health (8.5%), while only serving around 15% of the population. Private healthcare currently accounts for disproportionate availability of facilities as well as extreme misdistribution of pharmaceutical personnel between the private and public sector (Labonté et al., 2015; Meyer et al., 2017).

Furthermore, like many other countries in Africa, South Africa faces a shortage of skilled health workers, including physicians, nurses, dentists and pharmacists. The shortage of key human resources, together with the worsening burden of infectious disease in Sub Saharan Africa (including HIV, TB and malaria), is some of the challenges faced by the current healthcare system (Boschmans et al., 2015).

In 2011, the National Department of Health (NDoH) published their Quality Standards for Healthcare Establishments in South Africa, which describes the global development of quality improvement for healthcare facilities. The NDoH, also introduced the National Health Insurance (NHI) with the pilot phase that commenced in 2012 (National Department of He, 2015). The system will be dependent upon an adequate supply of pharmaceutical personnel, including pharmacists, pharmacist-assistants and pharmacy support personnel (3) The NHI will require the direct involvement of clinical pharmacists throughout the medication-use process, to ensure continuity of care, minimised risk and even reduced mortalities with an increasing improvement of patient outcomes (Labonté et al., 2015; Meyer et al., 2017). Pharmacists are required to play a role in patient safety, clinical governance and care by reducing adverse events caused by medication or medication errors. In this regard, an improvement of medication reconciliation services can improve medication safety (Labonté et al., 2015; Meyer et al., 2017). According to the Quality Standards for Healthcare Establishments, pharmacists must also ensure that medicines are readily available to patients (National Department of He, 2011).

Although the functions described in the Quality Standards for Healthcare Establishments (NDoH) are expected from pharmacists, South Africa has an estimated under-provision of pharmacists of around 60%, with only around 35% working in the institutional section (Labonté et al., 2015; Meyer et al., 2017). This leads to a burden on institutional pharmacists to provide pharmacy-based functions like dispensing and procurement. It may also lead to suboptimal clinical care, leaving the pharmacist with minimal or no time to perform ward-based functions like medication review and patient counselling, leading to suboptimal clinical care (South African Pharmacy Council, 2012).

In developed areas if the world, like Europe and the United States, clinical pharmacy are well established, and also include specialist services including clinical pharmacy in the emergency department and geriatric services (Morgan et al., 2018; Van der Linden et al., 2020). Although elsewhere in Africa, countries like Kenya and Nigeria offer training to clinical pharmacists, isolated clinical pharmacy workshops were only offered in South Africa in the early 1980’s (Summers, 1991). However, training for clinical pharmacists developed rapidly thereafter, making South Africa the continent leaders in clinical pharmacy. Globally, there are many differences in the way that pharmacists practice clinical pharmacy (Miller et al., 2011). For the purposes of this paper, clinical pharmacy can be defined as “a health science specialty that embodies the application, by pharmacists, of the scientific principles of pharmacology, toxicology pharmacokinetics and therapeutics to the care of patients,” (ACCP). Few hospitals in SA provide patient-specific services that frees up a clinical pharmacist from distributive and dispensing responsibilities (Pickette et al., 2010; Bronkhorst et al., 2018). In South Africa, the work climate in which clinical pharmacy is practiced is changing. Private hospital settings in South Africa have created posts for pharmacists, performing clinical oriented work, requiring a post-graduate degree in clinical pharmacy and preferably antimicrobial stewardship training. Pharmacists with postgraduate qualification in clinical pharmacy are limited, and private institutions make use of ward pharmacists to perform pharmaceutical care (Sasocp, 2013). Although South Africa is continually developing and evaluating programmes to assess use of medicines in the country and monitor the care of patients (Pickette et al., 2010; Bronkhorst et al., 2018), clinical pharmacy services across institutions cannot be generalised and requires further investigation.

The South African Pharmacy Council (SAPC) accepted the postgraduate curriculum that leads to specialist registration for clinical pharmacists, and postgraduate degree programmes with the aim to train clinical pharmacists are offered by universities (Pickette et al., 2010; Bronkhorst et al., 2018). However, it cannot be implemented until the registration of the specialisation is in place and accepted by the NDoH. Unfortunately, no standardised required level of education for practice purposes for clinical pharmacists are in place, which leads to different levels of pharmaceutical care offered in different institutions.

The difference between hospital pharmacy and clinical pharmacy as set out by the SAPC, lies in the involvement of the clinical pharmacist as a pharmaceutical partner in the multi professional health care team, as well as the role the clinical pharmacist must play in development and implementation of evidence based policies and procedures. The clinical pharmacist must also perform research and add to the academic community by publishing the research (South African Pharmacy Council (SAPC), 2010; South African Pharmacy Council, 2014).

To provide insight into the pressing issues of clinical pharmacy practice in South Africa, the paper sets out to contextualise the current profile of the pharmacists performing clinical functions as well as the different clinical functions performed in the public and private sector of the health care system.

**Key concepts.**

**Educational background:** In this study, educational background is seen as the highest qualification of the participant relating to pharmacy.
**Clinical Pharmacy:** can be defined as “a health science specialty that embodies the application, by pharmacists, of the scientific principles of pharmacology, toxicology pharmacokinetics and therapeutics to the care of patients” (ACCP)
**Clinical Pharmacist:** registered pharmacist trained in clinical aspects of patient care.
**Ward Pharmacist:** A registered pharmacist who becomes an integral and indispensable part of the professional health team of the hospital/institution.
**Pharmaceutical Care:** originally defined by Hepler and Strand as “the responsible provision of drug therapy for the purpose of achieving definite outcomes that improve a patient’s quality of life”.

## Materials and Methods

### Study Design and Duration

The study was an explorative, cross-sectional study, collecting quantitative data. The data collection period was 3 months.

### Study Population and Sample

The study population included all pharmacists who are performing clinical pharmacy or rendering pharmaceutical care duties with a patient-centred approach. As clinical pharmacy in SA is developing, few qualified clinical pharmacists are available in practise, rendering services in the in-patient setting. The study population was identified using purposive sampling from a previous study, which identified pharmacists rendering clinical services at ward level (14) and included 86 pharmacists from private as well as public tertiary hospitals from an estimated 110 practicing clinical pharmacists currently in SA (Bronkhorst et al., 2018). Private healthcare groups and NDoH provided e-mail addresses for potential participants, after obtaining ethical clearance.

### Data Collection Instrument

The scope of practice of a clinical pharmacist, and specific pharmaceutical care functions (Kaboli et al., 2006; South African Pharmacy Council, 2014) was used to develop a questionnaire on the different clinical functions a clinical pharmacist performs. A pilot test was performed with five eligible participants in the field, and data was not included in the results. The electronic platform Typeform™ was utilised to administer questionnaires and collect data. The questionnaire determined the level of qualification of the pharmacist performing pharmaceutical care, as well as the different functions performed at ward-level. The link was shared with participants *via* e-mail, and fortnightly follow-ups were made for a period of 2 months.

### Data Collection and Analysis

This questionnaire provided information on the level of education of pharmacists rendering clinical pharmacy services, either as a clinical pharmacist or as ward pharmacist. Different functions ward-based pharmacists perform as part of their duties were identified. Data was collated on an Excel^TM^ spreadsheet, and analysed quantitatively, to determine the number of pharmacists rendering pharmaceutical care, and the amount of time spent on these services.

Categorical data (e.g., demographic characteristics, qualifications, etc.) were summarised by frequency counts and percentage calculations with 95% confidence intervals. Continuous data (e.g., the years in practice and the time spend on ward activities) were summarised by mean values and standard deviations. Differences in continuous (percentages) data between public and private sector participants were compared by the Fisher Exact test. Pearson and Spearman correlation coefficients and 95% confidence intervals were calculated to determine differences between the private and public sectors. P-values less than 0.05 were considered significant. Bonferonni corrections were applied to p-values of public sector data, as the sample size were smaller than that for private sector. All statistical analyses were performed on SAS (SAS Institute Inc., Carey, NC, United States), Release 9.4.

## Results

### Participant Enrolment and Response Rate

**FIGURE 1:**
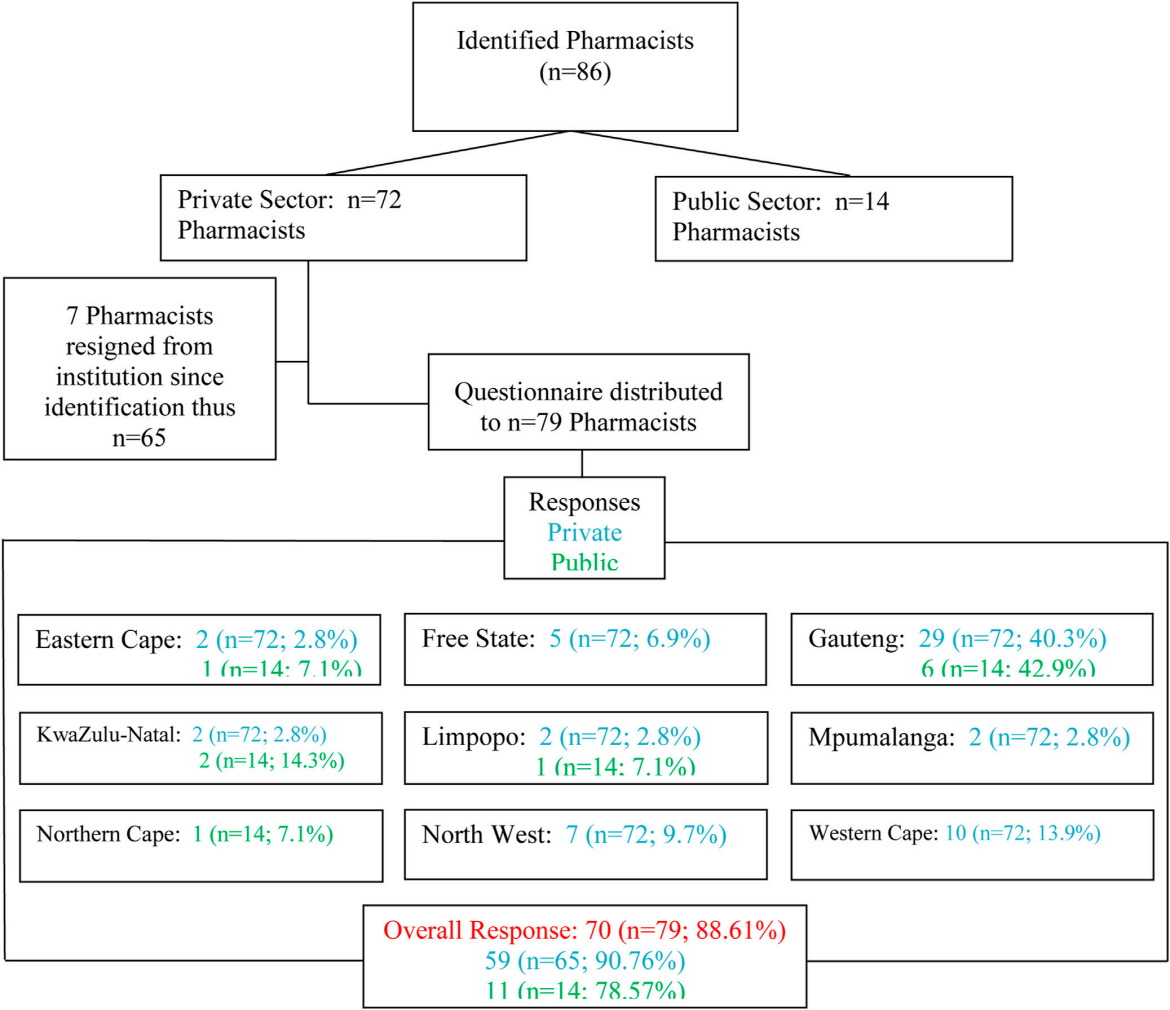
Number of respondents.

The sample size included 86 identified pharmacists from 130 hospitals, 72 (*n* = 79) in the private sector and 14 (*n* = 79) in the public sector. In the private sector, seven pharmacists resigned from the time of identification, thus the questionnaire was distributed to 65 pharmacists. Pharmacists were grouped according to geographic location and questionnaires were distributed electronically per province. A very good response rate for both the public and private sector was obtained; 59 (*n* = 65; 88.61%) and 11 (*n* = 14; 78.57%) respectively, resulting in an overall response rate of 88.61%. Figure 1 depicts the enrolment process and the study population as grouped geographically.

### Demographic Profile of Pharmacists Practicing Clinical Pharmacy.

Female participants represented 87.14% (*n* = 70) of the study population, while most participants (50%; *n* = 70) were in the age group 31–40 years of age and hold a BPharm degree (64.28%; *n* = 70). No statistical significance was found between demographic data of participants in the private sector and those in the public sector. More than half of pharmacists (54.29%; *n* = 70) reported that they spend only 1–2 hours per day in the ward doing clinical pharmacy activities. The demographic data of the participants are depicted in Table 1.

**TABLE 1:** Demographic Profile of the participants.

		Public	Private	Total	p-value^a^
Sex	Female	9 (12.86%)	52 (74.29%)	61 (87.14%)	0.6249
	Male	2 (2.86%)	7 (10.0%)	9 (12.86%)	
Age	20–30 years	3 (4.29%)	12 (17.14%)	15 (21.43%)	0.1909
	31–40 years	6 (8.57%)	29 (41.43%)	35 (50%)	
	41–50 years	2 (2.86%)	4 (5.71%)	6 (8.57%)	
	Older	0 (0%)	14 (20%)	14 (20%)	
Qualification	Dip Pharm	0 (0%)	1 (1.43%)	1 (1.43%)	0.0143
	BPharm	3 (4.29%)	42 (60.0%)	45 (64.28%)	
	MPharm/MSc(Med)	7 (10.0%)	14 (20.0%)	21 (30%)	
	PharmD/PhD	1 (1.43%)	1 (1.43%)	2 (2.86%)	
Societies	PSSA	6 (8.57%)	31 (44.29%)	37 (52.86%)	0.5347
	SAAHIP	3 (4.29%)	14 (20.0%)	17 (24.28%)	
	SASOCP	5 (7.14%)	33 (47.14%)	38 (54.29%)	
	Other	0 (0%)	12 (17.14%)	12 (17.14%)	
Years’ Experience	0–5 years	3 (4.29%)	11 (15.71%)	14 (20.00%)	0.0798
	6–10 years	2 (2.86%)	23 (32.86%)	25 (35.71%)	
	11–20 years	5 (7.14%)	9 (12.86%)	14 (20%)	
	More than 20 years	1 (1.43%)	16 (22.86%)	17 (24.29%)	
Years Clinical Experience	0–1 year	3 (4.29%)	16 (22.86%)	19 (27.14%)	0.7194
	2–3 years	6 (8.57%)	28 (40.0%)	34 (48.57%)	
	4–5 years	2 (2.86%)	7 (10.0%)	9 (12.86%)	
	More than 5 years	0 (0%)	8 (11.43%)	8 (11.43%)	
Hours spend in ward	1–2 h	9 (12.86%)	29 (41.43%)	38 (54.29%)	0.0891
	3–4 h	0 (0%)	18 (25.71%)	18 (25.71%)	
	4–6 h	0 (0%)	4 (5.71%)	4 (5.71%)	
	7–8 h	2 (2.86%)	9 (12.86%)	10 (14.29%)	

a Statistical significance were derived from using Fisher’s Exact test.

### Perceptions of Pharmacists Regarding Pharmaceutical Care and Educational Background

Most pharmacist in the private sector (32.2%; *n* = 59) agreed and in the public sector strongly agreed (45.45%; *n* = 11) that they have the necessary training to perform pharmaceutical care. The majority of respondents felt that interventions made by the pharmacist improved the rational use of medicine (47.46% in the private sector; *n* = 59 and 54.55% in the public sector; *n* = 11). They are of the opinion that their interventions can influence prescribing patterns in their institution (42.37% in the private sector; *n* = 59 and 63.63% in the public sector; *n* = 11) and that interventions may reduce polypharmacy (40.67% in the private sector; *n* = 59 and 54.55% in the public sector; *n* = 11). No statistical significance was detected between the opinions of pharmacists in the private and public sector (*p* = 0.2380–0.7615). Table 2 depicts the perceptions of pharmacists in the private and public sectors as well as the statistical significance between the opinions of the two groups.

**TABLE 2:** Perceptions of pharmacists.

	Strongly agree		Agree		Neutral		Disagree		Strongly disagree		p-value^a^
	Private	Public	Private	Public	Private	Public	Private	Public	Private	Public		
Do you have the necessary training to do your work?	13	5	19	2	11	1	13	1	5	0	0.4206
	22.03%	45.45%	32.20%	18.18%	18.64%	9.09%	22.03%	9.09%	8.47%	0%	
Interventions made by the pharmacist improved the rational use of medicine	28	6	16	2	5	0	5	1	5	2	0.7651
	47.46%	54.55%	27.12%	18.18%	8.47%	0%	8.47%	9.09%	8.47%	18.18%	
Interventions made by the pharmacists can influence prescribing patterns	25	7	18	1	8	1	4	0	4	2	0.3026
	42.37%	63.63	30.51%	9.09%	13.56%	9.09%	6.78%	0%	6.78%	18.18%	
Interventions made by the pharmacist can reduce the practice of polypharmacy	24	6	21	3	6	0	6	0	2	2	0.2380
	40.67%	54.55%	35.59%	27.27%	10.16%	0%	10.16%	0%	3.39%	18.18%	

a Statistical significance were derived from using the Fisher’s Exact test.

As shown in Table 3, no statistical significance (*p* = 0.662) could be found between the actual qualification held by participants and their opinion on whether they have the necessary qualification to perform clinical pharmacy functions. Participants who obtained a Master’s degree felt divided with five (*n* = 21) strongly agreeing that they have the necessary qualifications, and five disagreed.

**TABLE 3:** Opinion on Qualification compared to Qualification.

Opinion: Do you have the necessary qualification to perform clinical pharmacy functions?	BPharm^b^	MPharm/MSc(Med)	PharmD/PhD	Total	p-value^a^
Strongly Agree	11	5	1	17	*p* = 0.662
Agree	13	6		19	
Neutral	9	3		12	
Disagree	9	5		14	
Strongly Disagree	2	2	1	5	

a Statistical significance were derived from using Fisher’s Exact test with Bonferoni corrections.

b BPharm including enrolled for continued studies.

### Current Practice of Clinical Pharmacy

**FIGURE 2:**
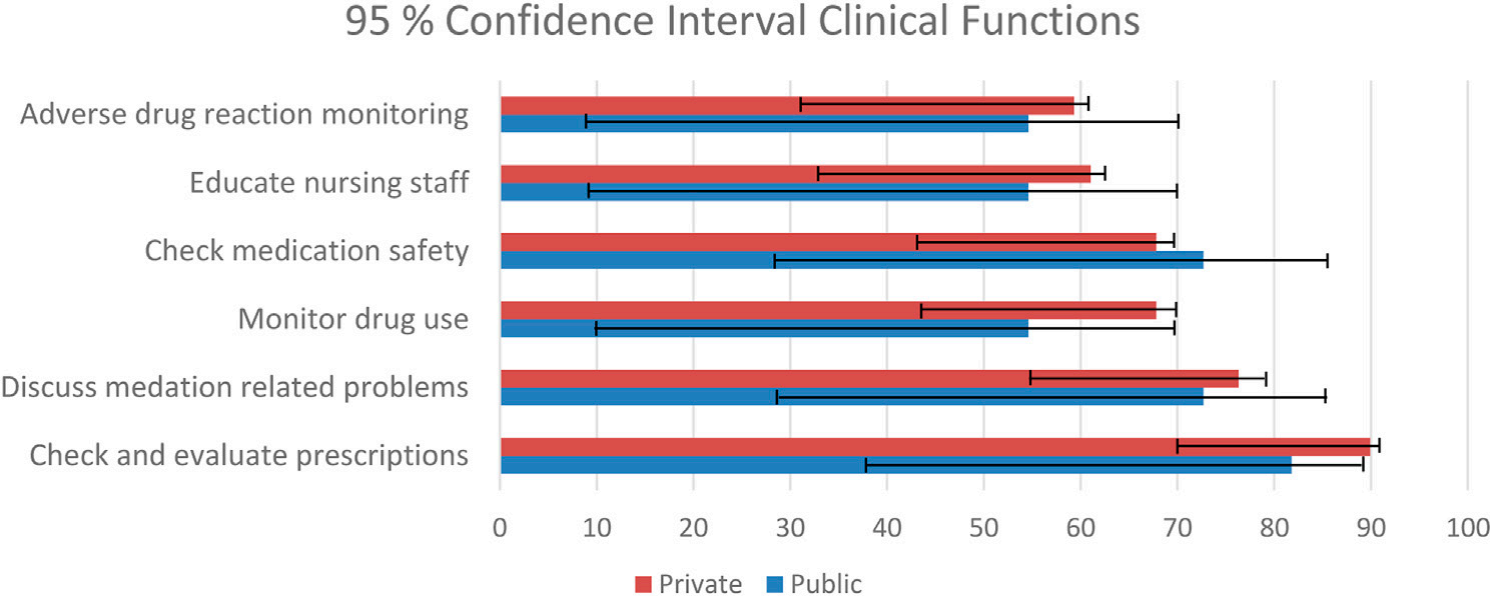
Clinical functions.

**FIGURE 3:**
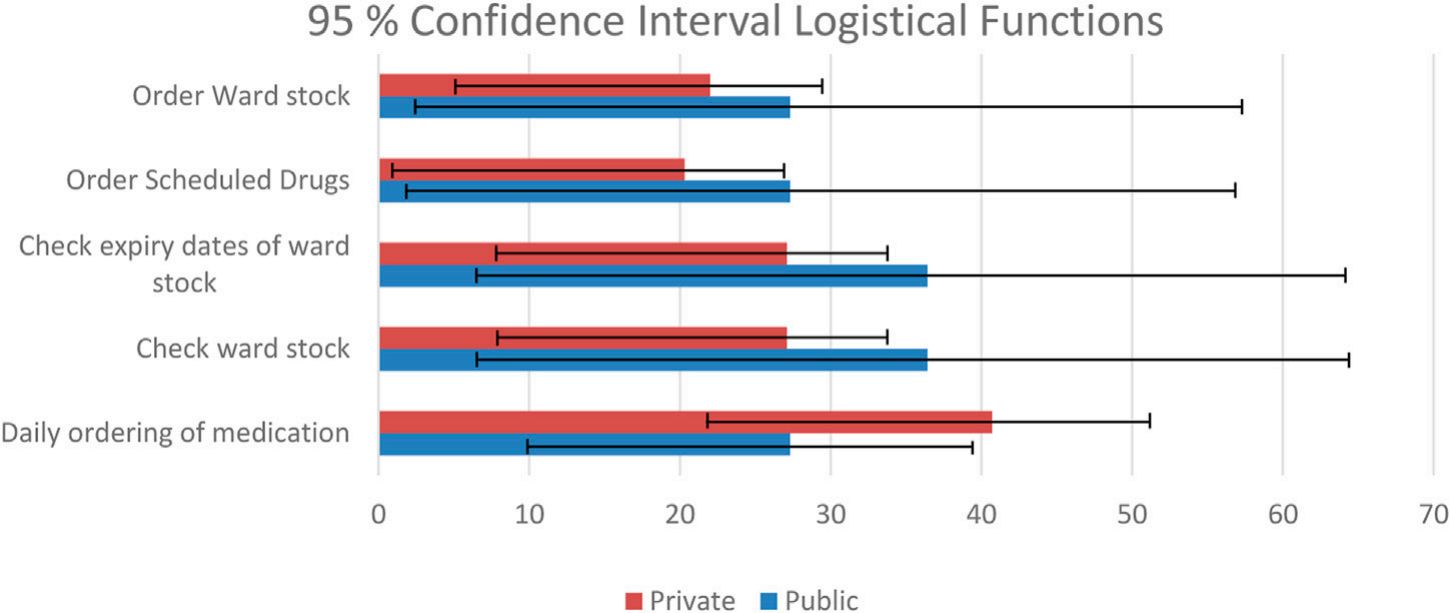
Logistical functions.

Pharmacists indicated the type of functions they perform in the ward setting. The functions were separated into clinical functions, which included adverse drug reaction monitoring, evaluating prescriptions, checking medication safety, discussing medication related problems with prescribers, education to nursing staff, doctors and patients, medication reconciliation, ward rounds and therapeutic drug monitoring. The logistical functions included checking expiry dates of ward stock, cytotoxic admixing, daily ordering of medication and ordering of ward stock and scheduled drugs. The clinical function that is performed most is the checking and evaluation of prescriptions (private sector 89.9% and the public sector 81.8%) while the top logistical function for the private sector is daily ordering of medication (40.7%), while in the public sector it is the checking of ward stock and expiry dates (36.4%). The top six clinical functions, with a 95% confidence interval is depicted in Figure 2, and the top five logistical functions, with a 95% confidence interval is depicted in Figure 3. The differences between the private and public sector are indicated.

## Discussion

In a previous study it was found that paper surveys achieved significantly higher (43.4 vs 43.4%33.7%) response rates compared to online surveys (Guo et al., 2016). Reasons stated for this phenomenon include factors like complicated login procedures, trouble with web navigation and unclear instructions. The response rate in this study was very high, compared to those stated in literature, possibly because the population was a secondary sample from a previous study (South African Pharmacy Council (SAPC), 2010; South African Pharmacy Council, 2014), purposively and a link to the questionnaire was provided via e-mail, which made the process easier. Furthermore, although motivation to participate in research are low (Adefuye et al., 2020), the high response rate is possible because of participants’ previous experience with research, as they possess a post-graduate degree.

The majority of the participants were female, and in the age group 20–30 years, which is representative of the pharmacy community in South Africa. The SAPC reported that around 62% of the pharmacy workforce comprises of women and the greater part of pharmacists in South Africa are younger, with most pharmacists falling in the age group below 35 years (Pharmacy Council, 2018).

Both in the public and private sectors, with staff-shortages more pronounced in public than in private institutional sectors (South African Pharmacy Council (SAPC), 2010; South African Pharmacy Council, 2014), the majority of pharmacists felt that provided more time, their involvement in ward-based pharmaceutical care can improve the rational use of medication, influence prescribing practice and reduce polypharmacy in their different settings. Furthermore, they felt that standardised positions with specialist certification, as found in the US, might enhance practice. Many studies around the world have been published to support this belief (De Jager et al., 2014; Francis and Abraham, 2014; Kim et al., 2015; Gorman and Slavik, 2016). Contrary to this a study performed in Australia, found clinical interventions provided by basic level pharmacists to be poorly coordinated, although effective (Buss et al., 2018).

From the findings of this study, the top clinical functions pharmacists spend time on include adverse drug reaction monitoring, evaluating prescriptions, checking medication safety, medication reconciliation, ward rounds and therapeutic drug monitoring. The role of clinical pharmacists in medication errors, adverse drug events, therapeutic drug monitoring and antimicrobial stewardship (Ali et al., 2013; Williams et al., 2013; Sebaaly et al., 2015; Truter et al., 2017; Van Kemseke et al., 2017; Schellack et al., 2018) have been described extensively, both internationally and nationally. In a study from Finland, it was noted that medication reconciliation increased with 63% with the increase of clinical pharmacy services in hospitals over a 5 year period, improving medication safety with 87% (Schepel et al., 2019). Furthermore, the role of the pharmacist in specialty settings like critical care, neonatology, cardiology and infectious diseases has been described (De Jager et al., 2014; Francis and Abraham, 2014; Kim et al., 2015; Gorman and Slavik, 2016). In developed countries it has also be expanded to describe the clinical pharmacists role in the emergency department, geriatrics, ambulatory care and recently, management of COVID-19 (Morgan et al., 2018; Paudyal et al., 2020; Van der Linden et al., 2020). In SA, these functions are described by the SAPC in their GPP (South African Pharmacy Council (SAPC), 2010) document, although not specifically at specialist level, managing specific patient groups. As the registration process for clinical pharmacists by the SAPC and NDoH are not finalised (Gous and Schellack, 2014), there exist a lack of specific policy regarding the functions to fulfil as a clinical pharmacist in SA.

In this study, the opinions of pharmacists did not differ regarding pharmaceutical practice, regardless of their highest qualification. The Master’s degree in Clinical Pharmacy offered by the Sefako Makgatho Health Sciences University (SMU), consist of a didactic modular component (2 years) and a research component (De Jager et al., 2014; Francis and Abraham, 2014; Kim et al., 2015; Gorman and Slavik, 2016). Many of the pharmacists participating in the study completed the modular component of their degree, but are still busy with the research component, hence did not obtain the degree yet. In a study performed in China, is was concluded that specific efforts must be made to improve pharmacist competence in order to improve pharmaceutical care (Xi et al., 2019). Differences between older and younger pharmacists, or longer experience with clinical work were also insignificant. This were different in a study done in Russia, that indicated that younger pharmacists (20–30 years old) do significantly fewer activities like prescription validation and evaluation of patient satisfaction than their older counterparts (Cordina et al., 2008). The study concluded that pharmacists in South Africa are performing a variety of functions at ward level, which include clinical functions as well as logistical functions.

This study can not be generalised to the general pharmacist population in South Africa. However, it is a good representation of clinical pharmacy services in South Africa, as it included the input from between 78 and 88% of pharmacists practicing clinical pharmacy in both public and private healthcare facilities.

## Strengths and Limitations

To the knowledge of the researcher, this is the first study in South Africa, which sought to engage pharmacists in pharmaceutical practice-based research on a large scale. A limitation was that the participants did not have the option to state if they were in the process of obtaining a Master’s degree, leading to indistinct clarification of qualification between the role of the dispensing pharmacists and pharmacists performing clinical functions.

## Recommendation

A certification system for qualified clinical pharmacists needs to be put in place, with an expectation of dedicated posts for clinical pharmacists. The influence of such a system, with dedicated posts for clinical pharmacists, may be evaluated retrospectively after implementation, to evaluate to influence of adequate time in the workday on the quality of clinical services provided to patients. The benefit of implementing a certification system will be to standardise the practice of clinical pharmacy services across different facilities, ensuring uniform delivery of clinical pharmacy practice. A future study on the perceptions of physicians, nurses and dispensing pharmacists will highlight the role of the clinical pharmacist in the multidisciplinary team.

## Conclusion

Many studies worldwide have shown the positive impact a clinical pharmacist can bring about in the healthcare setting. Even though clinical pharmacy in South Africa are rapidly developing, few pharmacists in positions are holding a MPharm degree in clinical pharmacy. Because of this fact, institutions are appointing pharmacists in the process of obtaining the degree. However, the pharmacists performing clinical functions at ward level perform numerous clinical functions, even if they spend only a small part of their workday in the wards. Around 90% of pharmacists doing clinical work evaluate prescriptions for medication errors in the ward setting. Pharmacists in South Africa are of the opinion that by doing clinical work, they reduce medication errors and improve prescribing patterns in their standard practices of clinical pharmacy. To compensate for the shortages of human resourses in pharmacy, pharmacists in clinical roles also fulfill some logistical functions like daily ordering of patient medication and ward-stock.

## Data Availability

The raw data supporting the conclusion of this article will be made available by the authors, without undue reservation.
